# Inquiries into the Origin and the Mode of Expulsion of the Ascaris Lumbricoides

**Published:** 1832

**Authors:** George R. Morton

**Affiliations:** Coshocton, Ohio


### Art. III.—*Inquiries into the Origin, and the Mode of Expulsion of the Ascaris Lombricoides.* By George R. Morton, M. D. Coshocton, Ohio.

When the important influence which helminthic diseases exercise upon the health of children is universally considered, it cannot but be acknowledged, that, with all our experience and skill, we are still lamentably ignorant both of their true nature, and of the most unfailing means of effecting their removal. The labored speculations which crowd our medical libraries furnish abundant evidence that our ignorance upon this subject is not owing to the want of attentive observation; but rather to the mysteries in which the subject is shrouded, and to our deficiency of information as to the peculiar habitudes of the ascaris itself. It is hence that such conflicting sentiments have arisen—such wild and untenable theories—and such inert and negative modes of practice. While one nosologist, as an advocate for the doctrine of "omnia ex ovo," is eagerly engaged in proving the developement of the worm in the human system from *ovula concealed in the ingesta*—another is equally sanguine to establish his favorite theory of its *spontaneous generation*. One distinguished writer arrives at the conclusion, that, "worms form part of a healthy constitution, and are scarcely injurious, but from accidental circumstances"—while another attributes to their irritation, a formidable list of the most distressing disorders. With views so utterly at variance, the difference in practice can readily be imagined;—the most temporizing, if not inert—the most active, if not absolutely poisonous.

It is not my intention in this article to assert a claim to any new or original ideas in regard to the origin of this isolated animal; but from the views I shall advance, a methodus medendi has been adopted, the correctness or fallacy of which is fearlessly left to the unerring test of experience.

Before giving our views of the treatment proper for the removal of these animals, I wish to offer a few remarks upon the condition of the alimentary canal most favorable to the production and existence of worms.

It is a well-authenticated fact, that the majority of cases of verminous diseases, affect children under the age of six or seven years; and mostly those of a cachetic and debilitated habit of body. In such children the digestive faculties are always much impaired, and, of course, the lining membrane of the whole canal, is in a more or less excited and irritated condition. In this state of irritability, substances taken into the stomach as food, are necessarily but imperfectly digested, and the unassimilated matter, if it does not absolutely heighten, serves at least to continue the original disorder. The tone of the alimentary canal is thus gradually more and more reduced below the healthy standard, until it reaches that condition in which the food, instead of passing through a pure and uninterrupted process of nutritive digestion, undergoes some species of decomposition, if not fermentation. It is when this stage of *infantile dyspepsia* (as it might be called,) has been arrived at, as evinced by flatulence, and by unhealthy and mal-digested foeces, that we are most likely to find the first appearance of the ascaris lumbricoides.

It will be remembered also, that this is a period of life at which the bowels are usually loaded with excess of mucus; and which of itself, in the habit I have described as peculiarly susceptible to helminthic disease, will run into the same decomposition or fermentation, or at least, serve as a nidus for the generation, growth, and sustenance of these parasitical vermin.

However much the assertion, that verminous diseases are occasionally epidemic, may be ridiculed or denied, there are so many concurrent testimonies in its favor, that the fact cannot longer be controverted. My own limited experience has fully confirmed me in the belief, that these affections are sometimes in a small degree, epidemic, or at least *endemic* to a considerable section of country.

The sympathetic influence mutually exercised between the alimentary canal and the skin, is sufficiently obvious, and well understood. When, therefore, after sudden atmospherical variations, particularly from a hot and dry state of the weather, to one chilly and laden with moisture, as is so common in our vernal months—many children are simultaneously afflicted with symptoms indicative of lumbricoides, can we attribute it rationally to any other cause than the one so apparent? Rejecting as unsatisfactory, all theories, how can we so plausibly account for the appearance of worms after sudden changes of air, as by that which refers the explanation, to a sympathetically depraved condition of the stomach and smaller intestines, and to a vitiated and perverted digestion.

We are perfectly conversant with the fact, that a spare and penurious diet, particularly of unripe vegetables and fruit, is one of the most frequent exciting causes of the production of lumbricoides in young children, and especially in those of the poor. It is perfectly immaterial whether such diet has undergone a culinary process or not, symptoms of worms are generally very certain to follow its continued employment. Independent of the injurious effect such unassimilating food must finally produce directly on the stomach and bowels, it is plain that it cannot long remain in the primæ viæ without undergoing fermentation, and consequently giving birth to these troublesome vermin.

That a diet perfectly devoid of stimulating and tonic ingredients, will give rise to worms in the human body, is susceptible of proof. I need mention but two instances in support of this position—both long embodied in our medical Text Books. Criminals, by an old law of Holland, "were frequently punished by being kept upon bread without salt." "The effect was most horrible; these wretched creatures having been devoured by worms engendered in their own stomachs." Again, it is familiar to every one acquainted with the peculiarities of the sheep, that the want of an adequate supply of salt, whether in substance or chemically combined in the vegetables of salt marshes, is sure to render the animal a prey to the fasciola or fluke—in common language, to the "rot."

The view which has just been taken of this interesting topic appears to me not only philosophical in theory; but I am convinced, perfectly rational and just in practice. I am aware that it is from the very nature of the subject, crude: yet I cannot refrain from expressing a hope that my suggestions may be carefully tested and examined.

Before passing on to the consideration of the plan of treatment I have deduced from these views, I would remark in brief, that the symptoms laid down in books, and particularly by Dr. Heberden, as characteristic and pathognomonic, when considered in detail, are at best but very fallacious. They are generally as indicative of *any other disease of the bowels* as they are of worms. I have seen most of the leading symptoms combined—the offensive breath—tumid abdomen—picking at the nose—

convulsions—voracity or loss of appetite—dry hacking cough; and yet a long continued course of anthelmintic remedies, afforded not the least trace of lumbricoides or any other worm; nothing in fine was apparent but a highly irritable condition of the mucous coat of the intestine, which such a course of medicines could not fail to increase. My own experience and observation have furnished me with *three diagnostie signs* which, when present with those enumerated, I can safely pronounce *unerring:* they are, *a pearly whiteness of the sclerotic coat of the eye—a brilliant shining carmine tinge of the lips, particularly of the upper*—and *a peculiar and almost indescribable depression of the alæ nasi.* This last appearance I can compare to nothing except that assumed by the same organ in the facies hippocratica.

Having thus expressed my opinions as to the probable origin and nature of the ascaris lumbricoides, it only remains to point out the principal indications of cure—and the manner by which those indications are most effectually subserved. It is acknowledged by Dr. Good, that "there is yet great space for improvement in the mode of treating the complaint;" and that "a decisive vermifuge process is yet a desideratum in medical practice." This uncertainty and defect in practice is evident from the empirical mode most physicians resort to for the removal of these disagreeable vermin, and may fairly be imputed to the unsettled ideas they have of the pathology of the disease. Previous to my adoption of the opinions I have explained in this article, I had found no disease to which children were peculiarly liable, more troublesome and vexatious in its remedial management than this. After a continued and perhaps successful use of some article of the numerous list of anthelmintics, I was but too frequently mortified by the gradually declining state of my little patients' health—and the recurrence of verminous symptoms in their original violence. Subsequent observation has satisfied me that this arose from the employment of *simple* and *uncombined* substances, whose only effect was to expel the worms, either by their mechanical or by their narcotic action; and of course, operating upon the debilitated and relaxed surface of the intestinal tube, to increase instead of removing that very condition which I consider to be the foundation of the disease.

The indications at which I now aim are, first to expel the worms, and then to remove that condition of the bowels which predisposes to and is, in fact, the proximate cause of invermination. It will be of little use to effect the expulsion of lumbricoides, if the tone of the alimentary canal is not specially altered. We but too frequently aim at removing the *apparent cause* of offence to the system, and congratulate ourselves prematurely on our success, without considering that the *lumbricoides* themselves are but the *effect* of a cause still more remote. We thus, in our anxiety to attain the first, and seemingly most important indication, neglect or disregard altogether the more imperious calls of the second. Before forming any definite opinion on the subject, by which to be guided, my early practice, I am free to confess, was not a little empirical. If one anthelmintic failed, I had but recourse to another till my object was obtained in the discharge of the vermin. My favorite remedies were, Ol. Chenopod. Anthel. dissolved in Spts. Ter.; a decoction of Spigelia Marilandica; or a dose of Calomel at night, followed in the morning by a purge after the following recipe:—

Rx. Fol. Senna, oz.ss
Spigelia, oz.ss
Manna, dr. ij.

to a pint of boiling water, of which, when cold, the patient partook freely until catharsis was effected. Yet even in these, though generally successful in expelling the worms, there was something unsatisfactory, as the cure was only temporary. Attentive consideration of the subject led me to test the utility of prescriptions, which would *combine the two leading objects at one and the same time*—the removal of the worms, and the restoration of the tone of the bowels. That I have finally fully succeeded in the discovery of such a combination, upwards of one hundred cases in which it has been employed without a single failure, have satisfactorily convinced me. Since the knowledge of this, I have used no other vermifuge whatever. The combination is formed after the following:—

Ol. Oliv, purif. oz.i.
" Chenopod. Anthel. m. 40,
" Anisi, oz.i,
' Templinum, oz.i,
Tinct. Myrrh Satur. oz.ss. m.

The three last named oils are to be added, when thoroughly mixed, to the Tincture, and perfectly combined, before being mixed with the Ol. Oliv. It is necessary that the Ol. Chenopo. Anth. be perfectly unadulterated. Otherwise the combination will be almost inert. The dose of this for a child of four years, is one drachm every hour until purgation is induced, and repeated daily for three days. In this prescription, we have in the ol. oliv, a mild, yet effectual purgative; in the ol. Chenop. Anth., an exceedingly bitter article, not only decidedly noxious to the worm, but considerably tonic; in the ol. Anisi, a placebo to the taste—a corrector of the acrimony of the Chenopod., and a carminative to allay spasmodic action; and the Ol. Templinum, (the distilled oil from the green cones of the pinus sylvestris,) a moderate vermifuge. One important advantage this combination possesses, independent of the facility with which it may be administered to the youngest infant, is, that a resort to any preparatory process, whether medicinal or dietetic, is altogether unnecessary. Even where the bowels are in an irritable condition, without the presence of worms, I have found it eminently useful.

In the early part of the article, I adverted to the opinion entertained by Dr. Parr, that worms are harmless tenants of the alimentary canal: as also to that expressed by various writers, that they excite a formidable list of diseases. To the former no serious reply is needed, for its weakness is self-evident; but to the latter, I can conscientiously subscribe. My own experience has satisfied me, that chorea, epilepsy, amaurosis, perverted vision, long protracted syncope, and imbecility of mind are among its frequent effects. But very lately, a case of convulsions, the severest I ever witnessed in a child, arose from this cause, and yielded, after the expulsion of a mass of lumbricoides by the vermifuge I mentioned above. A peculiar species of fever, with distinct exacerbations, and remissions and much gastric distress, I have frequently noticed—and have as frequently removed by the same remedy, when its appearance, in connexion with the diagnostic signs I have laid down, rendered me aware of its true helminthic character.

